# Devastating Neurological Outcome Following a Subarachnoid-Pleural Fistula During Thoracic Surgery

**DOI:** 10.7759/cureus.57838

**Published:** 2024-04-08

**Authors:** Benjamin Brueske, Christy He, Vikas Chauhan

**Affiliations:** 1 Anesthesiology, Columbia University Medical Center, New York, USA

**Keywords:** pneumonectomy, anesthesia, thoracic surgery, spontaneous pleural fistula, epidural

## Abstract

Pneumocephalus due to a subarachnoid-pleural fistula (SPF) has previously been described in the literature and is a rare complication following thoracic surgery. In this report, we discuss a patient who developed profound neurologic sequelae following right-sided pneumonectomy which was complicated by T2 nerve root avulsion and SPF development. The patient returned to the OR on postoperative day 21 in the setting of significant neurologic deterioration secondary to intracranial hypotension and pneumocephalus for SPF closure via thoracic laminectomy in the prone position. We present a rare cause of pneumocephalus and CSF leak, resulting in complications and sequelae and its management.

## Introduction

Subarachnoid-pleural fistula (SPF) is a rare but well-described complication of thoracic surgery [1-7]. Most patients recover with conservative management, but surgical intervention can be indicated if symptoms persist beyond one to two weeks [4]. Surgical treatment does help in improving the neurological function of many patients [2]. Here we present a rare case of SPF secondary to T2 nerve root avulsion after thoracotomy resulting in devastating neurological sequelae.

## Case presentation

**Figure 1:**
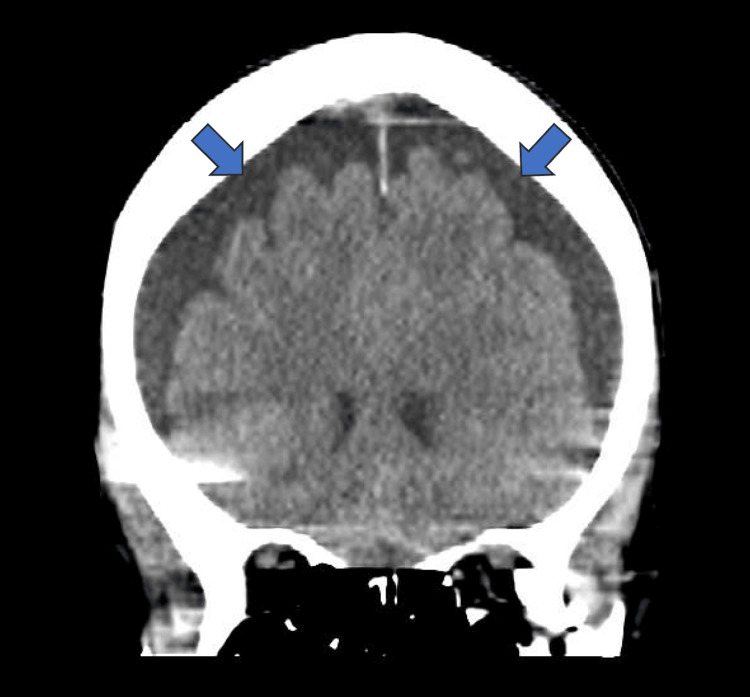
CT scan head showing pneumocephalus in the bilateral anterior convexity (arrows)

**Figure 2:**
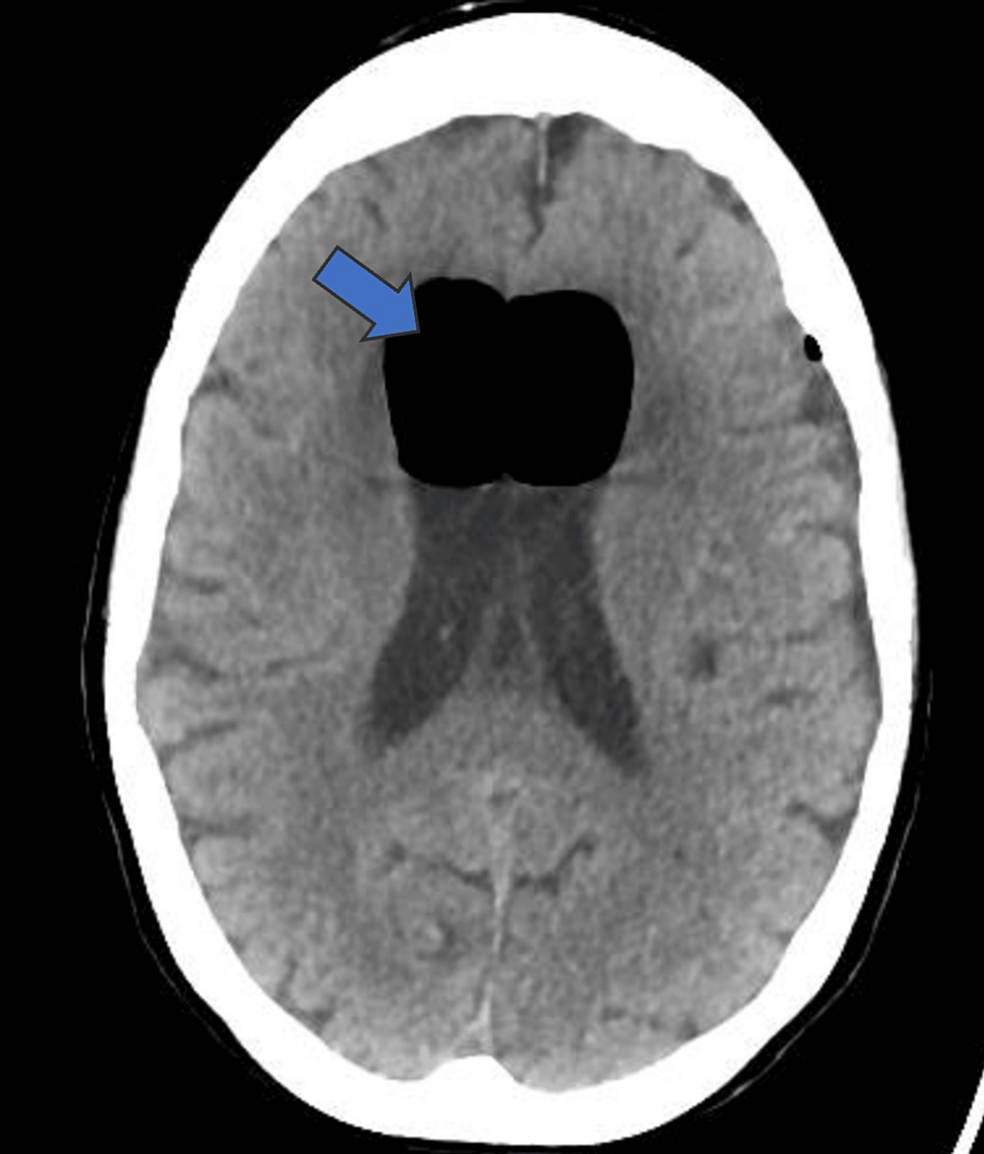
CT scan head showing pneumocephalus in the intraventricular system (arrow)

A 69-year-old female weighing 48 kilograms (Kg) presented for right-side complete pneumonectomy due to long-standing cavitary bronchiectasis secondary to repeat mycobacterial infection. The patient had a good baseline neurological function and was of American Society of Anesthesiology (ASA) Grade 4. Before induction of general anesthesia, she received a thoracic epidural for postoperative analgesia. An epidural catheter was placed at thoracic 5-6 spinal levels. There were three attempts made for the placement of the epidural needle and this was confirmed with loss of resistance to air. An epidural test dose was given which was negative. Standard ASA monitors (noninvasive blood pressure, oxygen saturation, electrocardiogram) were placed and general anesthesia was induced with fentanyl (2 microgram/kg), propofol (2 milligram/kg), and rocuronium (1 milligram/kg). This was followed by intubation with a single-lumen endotracheal tube that was then switched with a left-sided double-lumen endotracheal tube of size 35 (Sheridan^TM ^US) as needed for the surgery. An arterial line was placed, and adequate peripheral access was obtained. The surgical dissection was extensive and also required extra-pleural dissection secondary to chronic infection. Post-surgery, she was brought to the surgical intensive care unit with an endotracheal tube in situ. For postoperative pain, patient-controlled epidural analgesia was given with fentanyl 2 mcg/mL, bupivacaine 0.0625% in sodium chloride 0.9% 250 mL via the thoracic epidural. She was extubated on postoperative day one, uneventfully tube feeds started the following day and the patient had satisfactory neurological status. On postoperative day four, she developed facial asymmetry, unequal pupils, and altered mental status. A stroke code was called and she required endotracheal intubation. She was taken to the computer tomography (CT) scanner, which revealed pneumocephalus in the bilateral anterior convexity and intraventricular system (Figures 1, 2).

The thoracic epidural was removed on the same day and a chest tube was inserted. She was evaluated by neurosurgery and otolaryngology on the same day with no evidence of a cerebrospinal fluid (CSF) leak. She was given oxygen supplementation via a nonrebreather face mask. The neurological status recovered and a subsequent CT scan showed stable pneumocephalus. The patient required a tracheostomy for recurrent respiratory failure on postoperative day eight (twice in that period she was reintubated). Repeat imaging done on postoperative day nine was significant for persistent pneumocephalus and bilateral frontoparietal subdural hemorrhage suggesting a CSF leak. 

**Figure 3:**
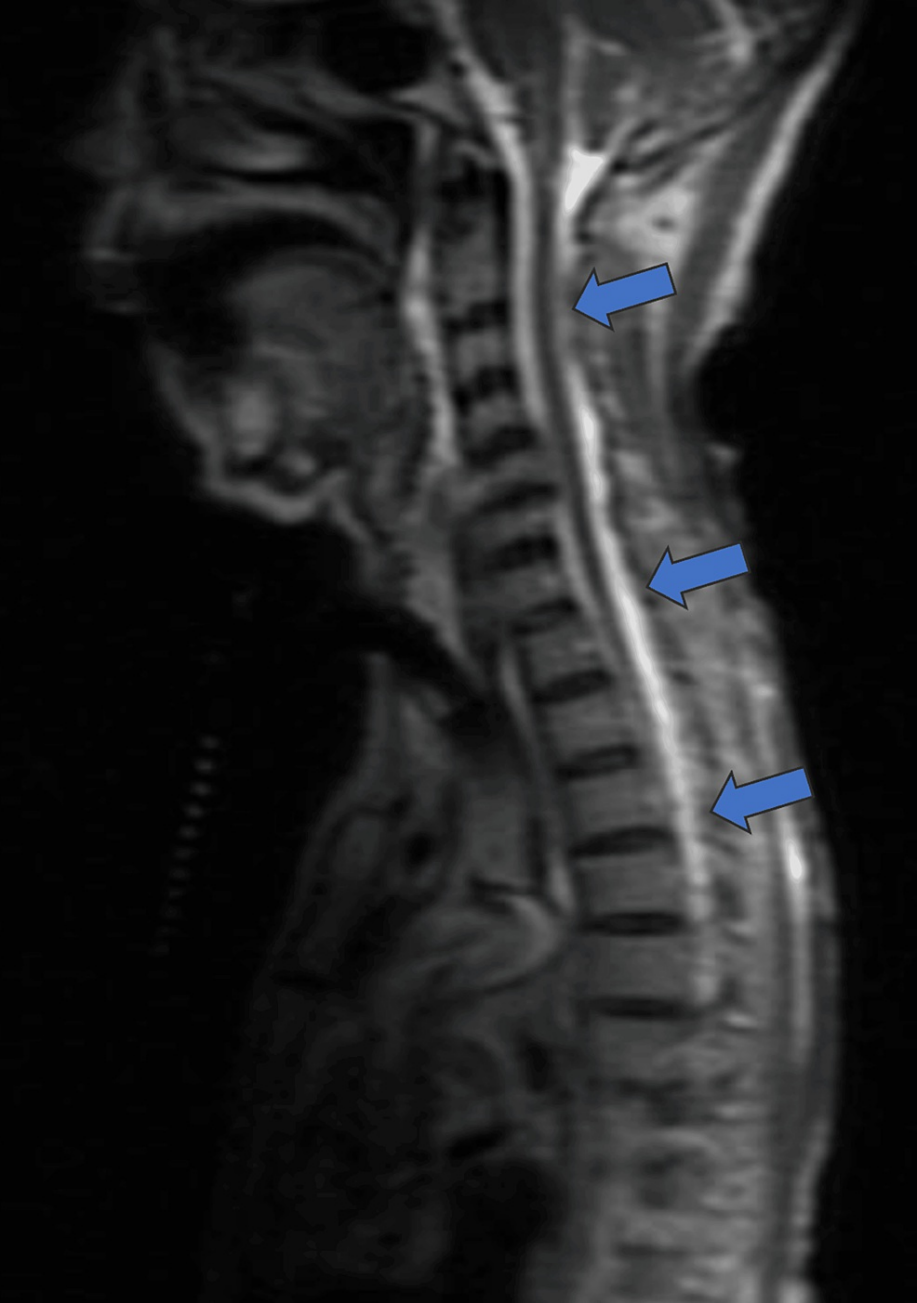
Magnetic resonance imaging (MRI) of spine showing spinal levels C3-T10 subdural collections (arrows)

Magnetic resonance imaging (MRI) of the spine was significant for C3-T10 subdural collections, also concerning CSF leak (Figure 3). MRI of the brain showed worsening subdural collections with associated mass effect. Also noted was a T2 nerve root avulsion that neurosurgery believed to be the likely cause of the CSF leak. This dural rent was thought to have formed a connection between the spinal canal and the thoracic cavity. Once the patient's condition was stable which included her following commands and moving all four limbs, she was transferred to the step-down unit. Immediately after transfer to the step-down unit, the patient’s neurological status deteriorated.

**Figure 4:**
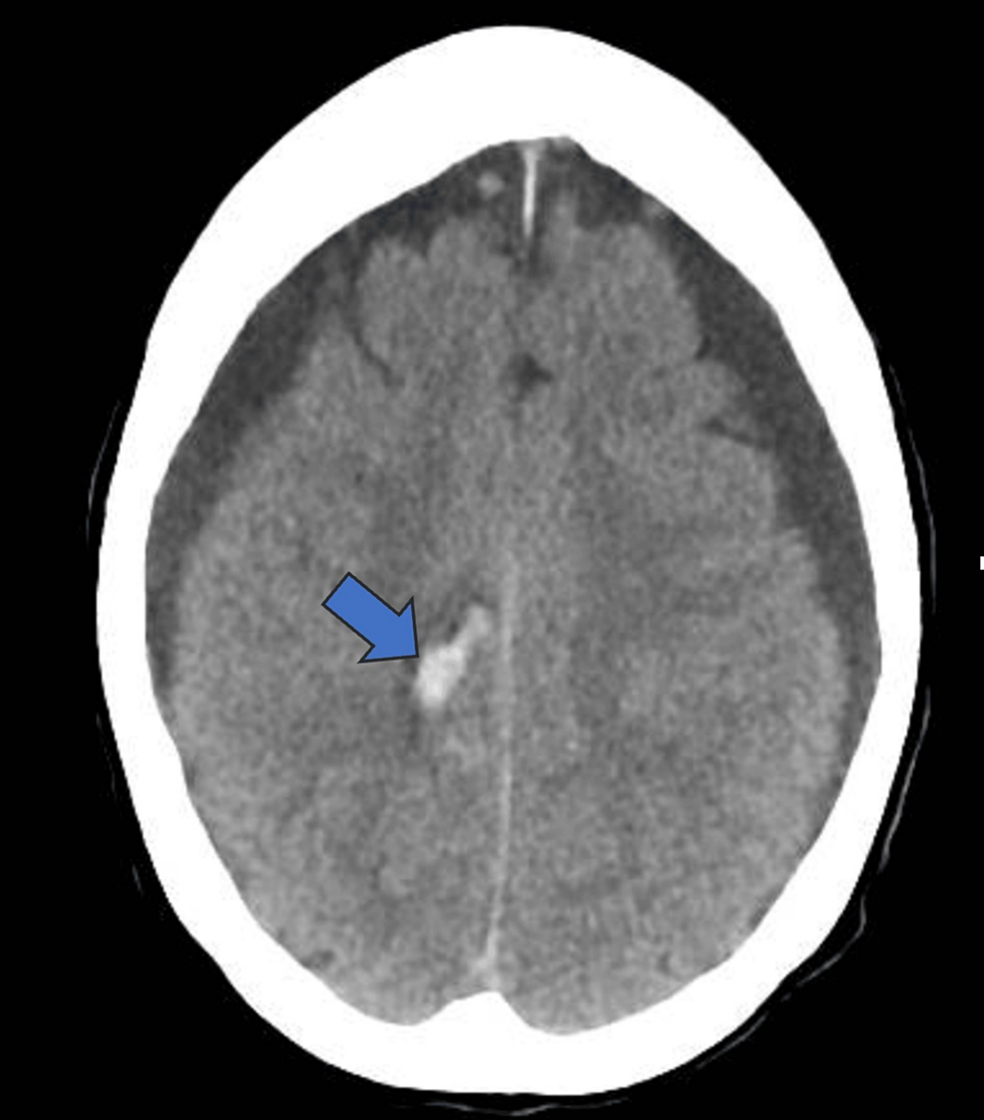
CT scan head showing intraparenchymal hemorrhage (arrow)

**Figure 5:**
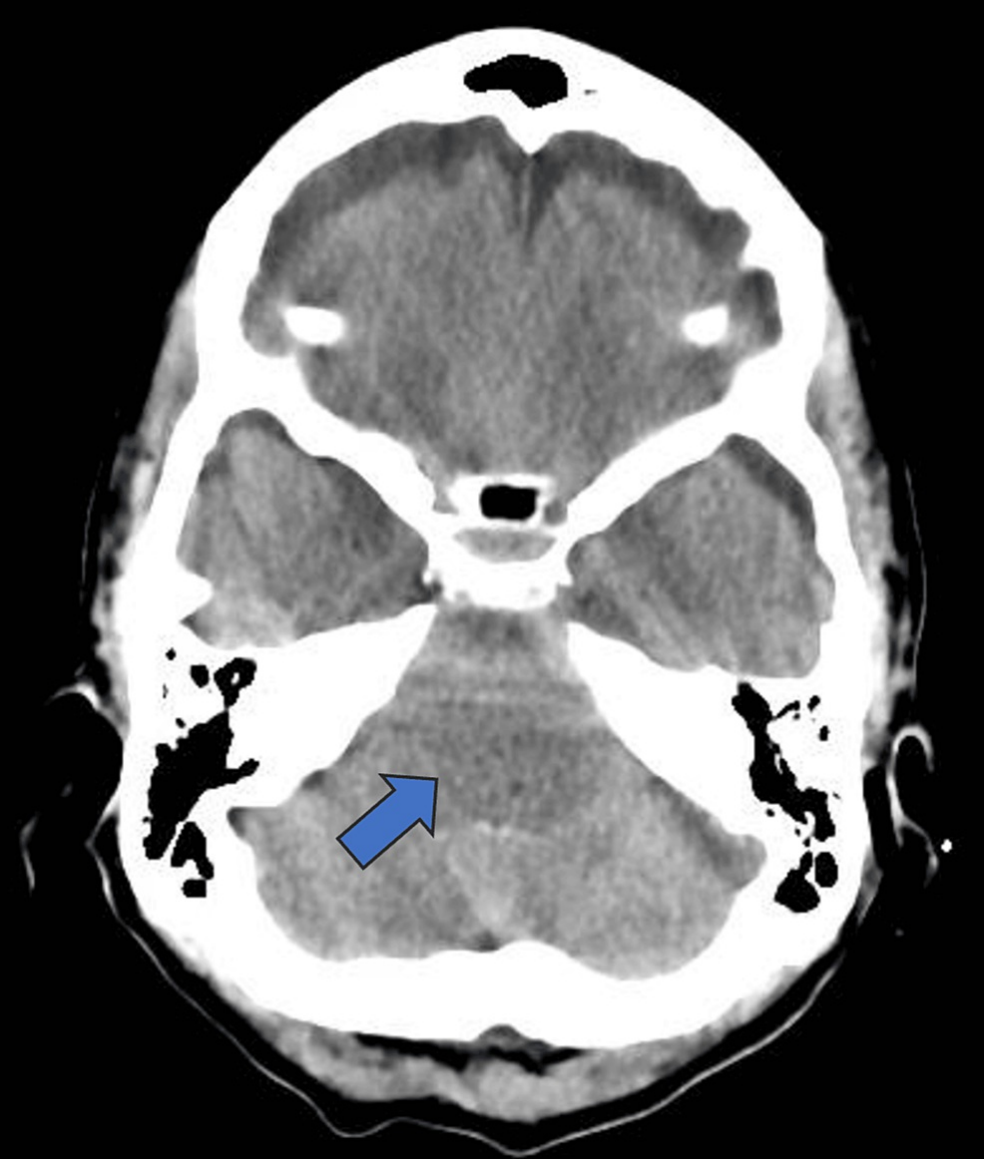
CT scan head showing effacement of basilar cisterns (arrow)

A CT scan revealed frontal acute intraparenchymal hemorrhage, worsening bilateral subdural collections, and effacement of basilar cisterns concerning brain herniation (Figure 4, 5). These suggested acutely elevated intracranial pressure. She was placed in a reverse Trendelenburg position to help reduce the risk of increased intracranial pressure and transferred to the neurological intensive care unit (NICU) and planned for laminectomy and dural repair with neurosurgery in the operating room. The patient had T wave inversions in the anterolateral leads on the electrocardiogram. A bedside transthoracic echocardiogram was performed which showed no regional wall motion abnormalities and the decision was made to proceed with surgery given the severity of her neurological status and that her EKG changes were likely due to her neurological changes and acutely raised intracranial pressure.

The patient was transported to the operating room with continuous monitoring of blood pressure, heart rate, and oxygenation. The patient was attached to the operating room ventilator, and tidal volume was adjusted to maintain peak inspiratory pressure <40cmH_2_O. Anesthetic induction was done with IV fentanyl (2 micrograms/kg), propofol (2 milligrams/kg), and rocuronium (1 milligram/kg). Gardner-Wells tongs were placed, and the patient was turned prone onto Jackson's table. Initial arterial blood gas demonstrated hypercapnia to 69 with metabolic compensation and pH of 7.32. The respiratory rate was increased to target normal pH, with a resolution of acidosis at a PaCO_2_ of 62. The patient was then maintained with a respiratory rate of 26 and a tidal volume of 270 ml. This was considered satisfactory given her post-pneumonectomy status and also her baseline respiratory physiology owing to long-term pulmonary pathology. The surgical team performed T1-T3 laminectomies and located an 8mm linear tear at the exit location of the right-sided T2 nerve root which was repaired with silk stitches. An opening was then identified through the T2-3 foramen into the lung cavity. The closure was performed with the placement of a conical bone fragment obtained during the laminectomy, which was tamped into the hole to seal the lung cavity from the epidural space, followed by a layer of tacky seal and facial closure. The patient tolerated the procedure well, with anesthesia maintained by propofol and fentanyl infusion. Electroencephalogram (EEG) was monitored throughout the procedure and demonstrated spectral edge frequencies <10. After closure, the patient was transferred to a supine position on the hospital bed and transported uneventfully to the NICU.

Postoperatively, her mental status remained poor. She was placed on EEG monitoring to evaluate for seizures as a cause for her poor mental status. EEG showed no seizure activity but was significant for severe global cerebral dysfunction. Around 12 weeks after her first thoracic surgery, the patient deceased.

## Discussion

Pneumocephalus is most commonly a result of trauma, surgery on the cranium, or surgery on the spine [4]. It is also a potential complication of epidural anesthesia, although postepidural pneumocephalus has only been described when loss of air resistance was used to identify epidural space [5,8]. In most cases, imaging reveals a small volume of air that resorbs after a few days [5]. Pneumocephalus can be characterized as simple pneumocephalus or tension pneumocephalus. Simple pneumocephalus is a benign accumulation of intracranial air without an increase in intracranial pressure, brain herniation, or compression of brain parenchyma. Tension pneumocephalus is an accumulation of intracerebral air leading to a significant increase in intracranial pressure with associated brain herniation [8,9]. Almost all cases of pneumocephalus from accidental dural puncture after epidural are simple pneumocephalus [8]. Symptoms are usually severe and may occur suddenly or within 72 hours of dural puncture (acute pneumocephalus), or greater than 72 hours after dural puncture (delayed pneumocephalus) [8]. In most cases of simple pneumocephalus spontaneous absorption of air is seen and symptom resolution after conservative management occurs within two to three weeks [10]. In our case, the epidural initially was thought to be the cause of pneumocephalus. There were no signs of dural puncture during the epidural placement, though there were three attempts all with loss of resistance to air. However, the persistence of symptoms after epidural removal, the large volume of intracranial air causing tension pneumocephalus, and the presence of a defect in the dural sheath where the initial dissection was done make the epidural a less likely cause of her presentation.

In thoracic surgery, pneumocephalus can occur due to dura matter damage if dissection is close to a neurovascular bundle emerging from the spine, during lymph node removal near the sympathetic chain, or in situations where excision of vertebrae is required [4,5]. When encountered later in the postoperative period, the most common explanation of pneumocephalus is traction and avulsion of the dorsal nerve root as it emerges from the spinal canal, which can be seen after forceful rib retraction [1,4]. A communication between the subarachnoid space and the pleural cavity is known as a SPF [1]. Unrecognized dural and parietal pleural injury can result in postoperative pleural effusion, intracranial hypotension, meningitis, or pneumocephalus [1].

Patients with an SPF typically present with neurologic symptoms including headache, altered mental status, or declining consciousness, as well as pulmonary symptoms including large transudative pleural effusions [1]. Symptoms typically appear on postoperative days 4 to 6, but delayed onset can occur up to two months postoperatively [2,6]. Our patient’s presentation was consistent with these findings.

Conservative therapy with hyperhydration and dorsal decubitus position often results in the resolution of symptoms. Surgical intervention is indicated if symptoms persist beyond one to two weeks with conservative management [4,5]. Surgical repair of the fistula is determined by the size and location of the defect, with laminectomy being preferred to repeat thoracotomy for initial exposure of the defect, and primary surgical closure with omental, fat, or muscle patch reinforcement having been reported [1].

Chest tube drainage can improve but also worsen this communication. Symptoms can worsen after chest tube removal when intrapleural air can extend under positive pressure into the subarachnoid space [2]. Chest tube drainage of the initial pleural effusion has demonstrated therapeutic benefit provided suction is not applied. The application of suction to chest tubes can promote continuous loss of cerebrospinal fluid and maintain patency of the subarachnoid-pleural fistula. For this reason, it is recommended to place chest tubes to water-seal with gravity-dependent drainage [1]. In our case, the chest tube was removed early in the postoperative period.

## Conclusions

Pneumocephalus may be attributable to a wide variety of causes, and a thorough workup is necessary to ensure appropriate treatment. This case highlights a rare mechanism of pneumocephalus which may be considered in patients postcardiothoracic surgery and emphasizes the importance of a broad differential diagnosis and a multidisciplinary approach to the diagnosis and management of patients with severe pneumocephalus.
